# Early and Long-Term Results of Stent Implantation for Aortic Coarctation in Pediatric Patients Compared to Adolescents: A Single Center Experience

**DOI:** 10.1155/2016/4818307

**Published:** 2016-01-27

**Authors:** Sara Bondanza, Maria Grazia Calevo, Maurizio Marasini

**Affiliations:** ^1^Cardiovascular Department, Giannina Gaslini Institute, 16148 Genoa, Italy; ^2^UOSD Epidemiology, Biostatistics and Committees, Giannina Gaslini Institute, 16148 Genoa, Italy

## Abstract

*Background*. Stents have become the treatment of choice for native aortic coarctation in adults and adolescents, but in pediatric patients insufficient data are currently available to identify the best therapeutic option.* Methods*. To compare the outcomes of pediatric and adolescent patients, we retrospectively evaluated early and long-term results of stenting for aortic coarctation in 34 patients divided into 2 groups (A and B) composed, respectively, of 17 children (mean age 8.2 ± 2.3, weight ≤30 kg) and 17 adolescents (mean age 14.3 ± 1.7, weight >30 kg).* Results*. No significant differences in outcome were found between groups immediately after the procedure. In all of our patients, peak systolic gradient pressure significantly decreased after stenting from 43.7 ± 12 to 1.7 ± 3.1 mmHg in group A and from 39.4 ± 16.8 to 1.6 ± 3 in group B (*p* < 0.0001). We observed early and late adverse events in both groups: early femoral vessel injury or thrombosis was more frequent in younger patients, as well as restenosis due to vessel growth requiring stent redilatations, often complicated by stent fractures. Data from long-term follow-up showed that, in younger patients, stress-related hypertension was more frequent.* Conclusions*. The procedure was immediately safe and effective in both groups. Pediatric patients must be accurately selected before stenting because they could probably need reinterventions and stents could impact on their future therapeutic perspectives.

## 1. Introduction

Although transcatheter stent implantation for aortic coarctation (AoCo) is increasingly used as a treatment option at younger ages, limited information is available on long-term results and follow-up in the pediatric population.

At present, surgical treatment of native AoCo is considered the first choice for younger patients (from neonates to children aged 12–18 months), while stenting is considered the preferable treatment for adolescents and adults [1]. For patients aged between 12–18 months and 9–11 years (usually the age when a child reaches a weight of 30 kg), there are currently insufficient data to determine the better therapeutic option between surgical and endovascular interventions, including balloon angioplasty or stenting [2].

We retrospectively compared early and long-term outcomes in a cohort of children and adolescents who underwent stenting for aortic coarctation in order to evaluate the safety and effectiveness of this procedure in younger patients.

## 2. Materials and Methods

### 2.1. Patient Population and Procedure Technique

**Figure 1:**
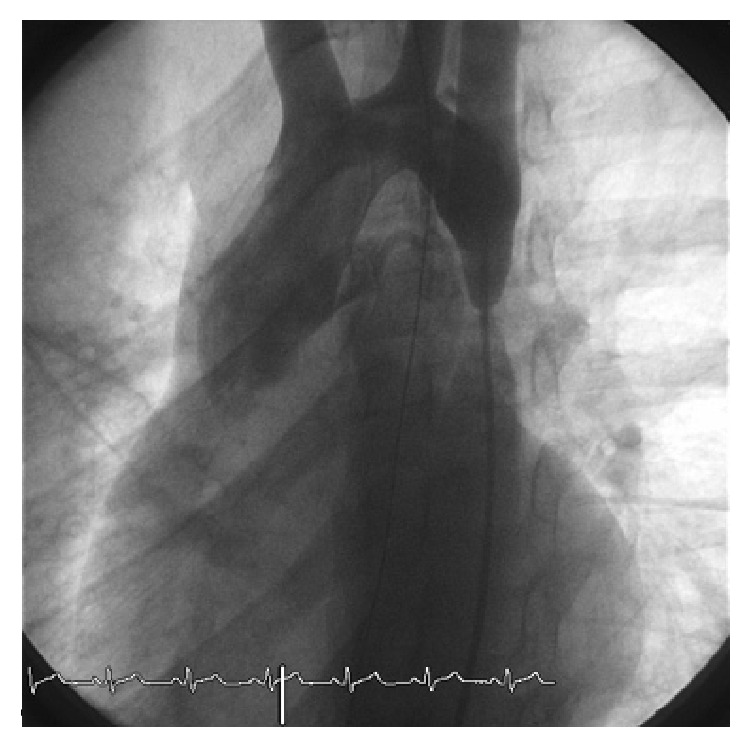
Anteroposterior view: severe aortic coarctation.

**Figure 2:**
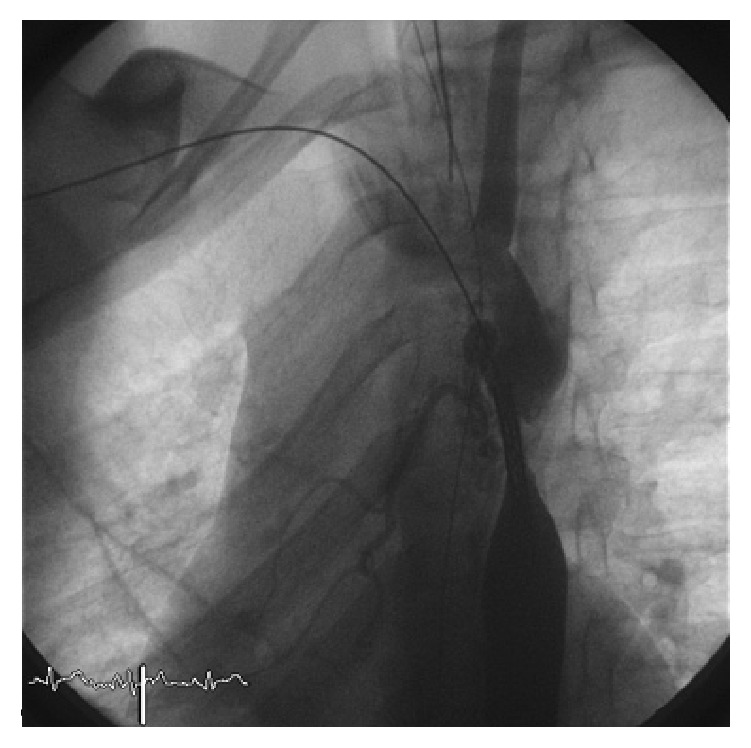
30° left anterior oblique view: stent positioning across the stenosis.

**Figure 3:**
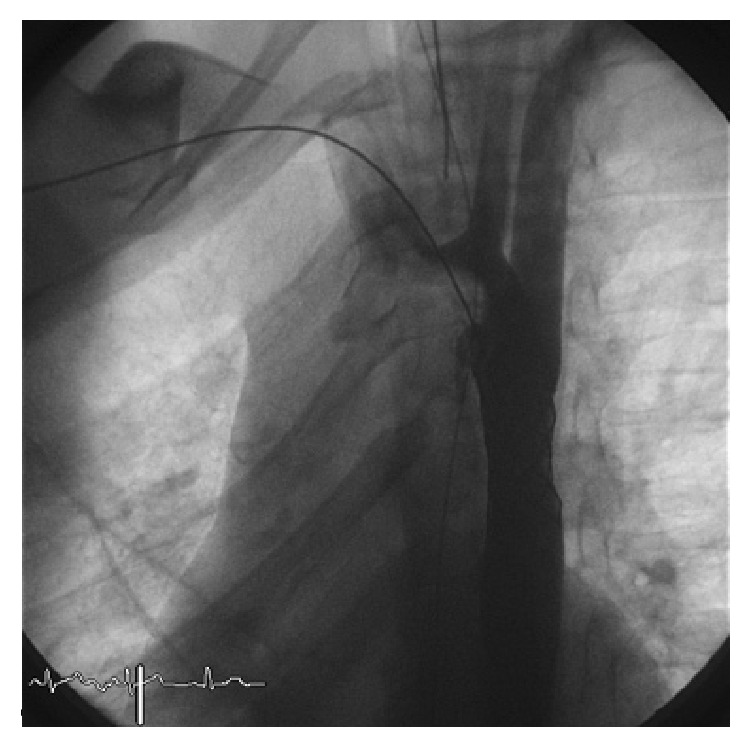
30° left anterior oblique view: stent successfully deployed.

We performed a retrospective analysis of early and long-term outcomes (follow-up until 10 years) of 34 consecutive patients who underwent stenting for aortic coarctation in the period between January 2000 and December 2014 in our institution. Inclusion criteria were patients aged between 18 months and 18 years with significant coarctation of the aorta and transcatheter systolic gradient >20 mmHg. Exclusion criteria were complex anatomy such as transverse arch or long isthmus hypoplasia which were surgically treated. In all of them, the procedure was performed under general anesthesia, after obtaining informed consent, according to the technique previously described in other studies [3, 4]. Femoral artery and vein access was obtained. The venous access was needed for the insertion of a temporary pacemaker in order to obtain a rapid right ventricle pacing and a consequent reduction of the cardiac output during stent deployment. Anticoagulation with heparin was maintained to keep the activated clotting time >200 seconds during the procedure. A 5-6 Fr sheath was positioned to perform the hemodynamic study. The narrow segment was crossed in a retrograde manner by the wire and the gradient measurement of the stenotic part and biplane angiography were performed to obtain the correct measurement (Figure 1). The tip of the wire was positioned into the subclavian artery or into the ascending aorta and a long sheath (Mullins, Medtronic, Minneapolis, MN) was pushed across the coarctation. The balloon diameter was the same as or 1 mm shorter than the diameter of the smallest aortic segment adjacent to the lesion. After choosing balloon and stent of proper size and type, stenting was performed under fluoroscopy and angiographic control (Figures 2 and 3). After stent positioning, hemodynamic study and angiography were repeated to confirm the success of the procedure and to detect possible complications. At the end of the procedure, hemostasis was mostly performed by manual compression; in some cases a Perclose (Abbott Vascular, Santa Clara, CA) was employed after local angiography of the femoral arteries. No carotid or brachial access was needed to cross the stenosis from above.

### 2.2. Outcomes

Immediate (IAE) and late adverse events (LAE) were chosen as primary outcomes. IAE included hospital mortality, thrombosis or injury of the femoral arteries, immediate stent migration, aortic dissection, and thrombosis of other arteries. LAE included restenosis, stent fracture or aneurysm after redilatation, stent migration, aortic dissection, femoral artery stenosis, or occlusion.

In order to compare the outcomes in children and adolescents and to evaluate the safety and effectiveness of stent implantation, we divided our patients into two groups: group A, including 17 patients aged between 3 and 11 years (mean 8.2 ± 2.3) and weighing ≤30 kg, and group B, including 17 patients aged between 12 and 18 years (mean 14.3 ± 1.7) and weighing >30 kg.

### 2.3. Statistical Analysis

Data are described as absolute and relative frequencies for categorical variables, while means, standard deviation (SD), medians, and range are used for continuous variables. Categorical data were compared by chi-square test or by Fisher's exact test in case of expected frequencies <5. Comparisons of quantitative variables between the 2 groups were performed by Student's *t*-test and *t*-test for paired data and Mann-Whitney *U* test. A *p* value less than 0.05 was considered statistically significant, and all *p* values were based upon two-tailed tests. Statistical analysis was performed using SPSS for Windows (SPSS Inc., Chicago, IL, USA).

## 3. Results

Our cohort included 34 patients: 24 males (70.6%) and 10 females (29.4%) aged between 3 and 18 years with significant aortic coarctation (AoCo). In our series, 23 (67.6%) patients were treated for isolated native AoCo, 10 (58.8%) in group A and 13 (76.5%) in group B, and 11 (32.3%) for recurrent coarctation after surgery or balloon dilatation, 7 (41.2%) in group A and 4 (23.5%) in group B. Mean systolic blood pressure before the procedure was similar in the two groups: 127 ± 12.5 mmHg and 137 ± 21 mmHg in groups A and B, respectively (*p* = 0.11). We implanted 5 Palmaz Stents (Johnson & Johnson International Systems, Warren, NJ), 4 in group A and 1 in group B, and 14 Cheatham Platinum (CP) stents (NuMED, Hopkinton, NY), 5 in group A and 9 in group B. In 2 patients of group B, we positioned two CP stents sequentially to cover long lesions. Finally, 17 covered CP stents (NuMED, Hopkinton, NY) were implanted (8 in group A and 9 in group B).

Demographic, preoperative, and operative data are summarized in Table 1.

In all of our patients, after stenting, the peak systolic gradient pressure significantly decreased from 43.7 ± 12 to 1.7 ± 3.1 mmHg in group A (*p* ≤ 0.0001) and from 39.4 ± 16.8 to 1.6 ± 3 in group B (*p* ≤ 0.0001).

We observed no early deaths during or immediately after the procedure. We observed 10 IAE (immediately to 24 hours after the procedure) in 6 (35.3%) patients of group A and in 4 (23.5%) of group B (*p* = 0.45). In group A, we observed 3 femoral artery injuries requiring immediate surgical repair with patch and 2 thrombosis of the femoral artery treated with heparin infusion. In one case, dissection after balloon angioplasty occurred and stenting of the lesion was mandatory. In group B, we observed 1 migration of the stent across the aortic arch requiring immediate stent removal and surgical repair of the aortic arch; 1 thrombosis of the hypoglossal artery treated with stent implantation; 1 thrombosis of the femoral artery treated with Fogarty thrombectomy; and 1 femoral artery injury treated with surgical patch repair. The most common immediate adverse events were femoral access problems, especially in the group of younger patients, but they were not statistically significant compared to those occurring in the group of patients weighing >30 kg (*p* = 0.39). At follow-up, we observed some late adverse events (2 to 10 years from the procedure) in both groups, 8 (47.1%) in group A and 5 (29.4%) in group B (*p* = 0.29). In pediatric patients (group A), we found 5 (29,4%) restenoses related to vessel growth, 4 treated successfully with redilatation of the stents (in 3 cases followed by fracture and shortening of the stent and in 1 by a small aneurysm) and 1 with surgery of the restenosis. We also found 1 migration of the stent to the distal part of the aortic arch requiring surgical repair and 2 femoral artery occlusions. In older patients (group B), we had 1 (5.8%) intrastent restenosis followed by stent redilatation and fracture 5 years later. In 2 cases, we also observed stent displacement (both were bare stents) in abdominal aorta; in the absence of clinical problems, stents were left in place. One small abdominal aorta dissection occurred with no clinical manifestations and one femoral artery stenosis was surgically treated. No late aneurysms were detected in any of our patients. We found restenosis due to vessel growth in 5 of our 17 pediatric patients and in 1 of the older patients (29.4% versus 5.8%), but no significant *p* values were detected (*p* = 0.45). Clinical evaluation was performed at 1 and 6 months after the procedure and subsequently annually in our ambulatory care service. Blood pressure with nonmercury devices was measured at right arm and at the right lower limb in order to detect possible pressure gradient. At clinical follow-up at 1 year from the procedure, we collected data about 24 patients, 12 of group A and 12 of group B. At baseline, no patients were under antihypertensive therapy. The mean systolic blood pressure in the two groups was, respectively, 120 ± 9 mmHg and 126.7 ± 16 mmHg (*p* = 0.22); 9 patients received medical treatment with ACE inhibitors (4 in group A and 5 in group B). After 5 years, we had data about 16 patients (8 of group A and 8 of group B): mean systolic blood pressure was 128 ± 16.5 mmHg and 128 ± 11 mmHg, respectively; 4 patients of group A and 2 of group B were under medical treatment (ACE inhibitors or beta blockers). After 10 years, data about 7 patients were available: 5 of group A, 3 with systemic hypertension successfully controlled with ACE inhibitors, and 2 of group B, who had normal systolic pressure values. Mean blood pressure values were 116 ± 6 and 128 ± 2 mmHg in groups A and B, respectively (Table 2). Twelve of all our patients (6 of group A and 6 of group B) had a treadmill stress test, according to the standard Bruce protocol, at 1 to 5 years from the procedure. Three patients of group A (50%) and 5 (83.3%) of group B had a normal response to the test and 3 (50%) of group A and 1 (16.7%) of group B had hypertension at peak exercise. At long-term follow-up, younger patients seemed to have more commonly stress hypertension, but the sample is too small and the statistics are not significant; therefore no definitive conclusions can be drawn.

## 4. Discussion

Intravascular stent implantation for the treatment of native or recurrent aortic coarctation yielded excellent short and intermediate term results [5].

In all of our patients, the peak systolic gradient pressure significantly decreased after stenting (*p* ≤ 0.0001) and we observed no early deaths during or immediately after the procedure. Therefore, in our experience the procedure proved to be immediately safe and effective; however early and long-term follow-up showed that adverse events can occur also many years after the procedure.

The limitations of our study are the single center experience, the retrospective analysis, the small size of our cohort of patients, and the limited amount of available follow-up data due to the difficulty in collecting information in the long term. For these reasons, the data obtained provide us with a trend and do not allow us to draw definitive conclusions.

**Figure 4:**
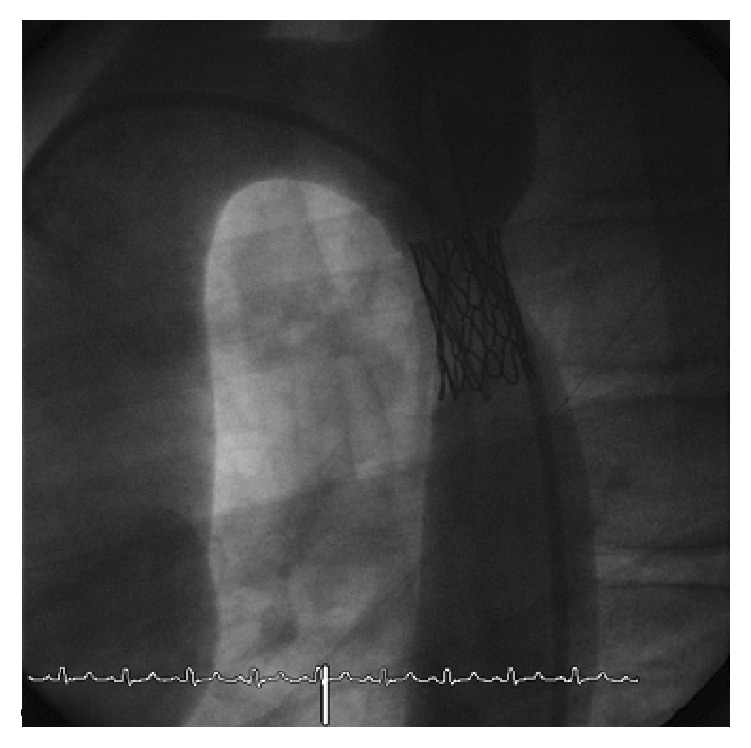
Lateral view: growth-induced narrowing of the previously implanted stent.

**Figure 5:**
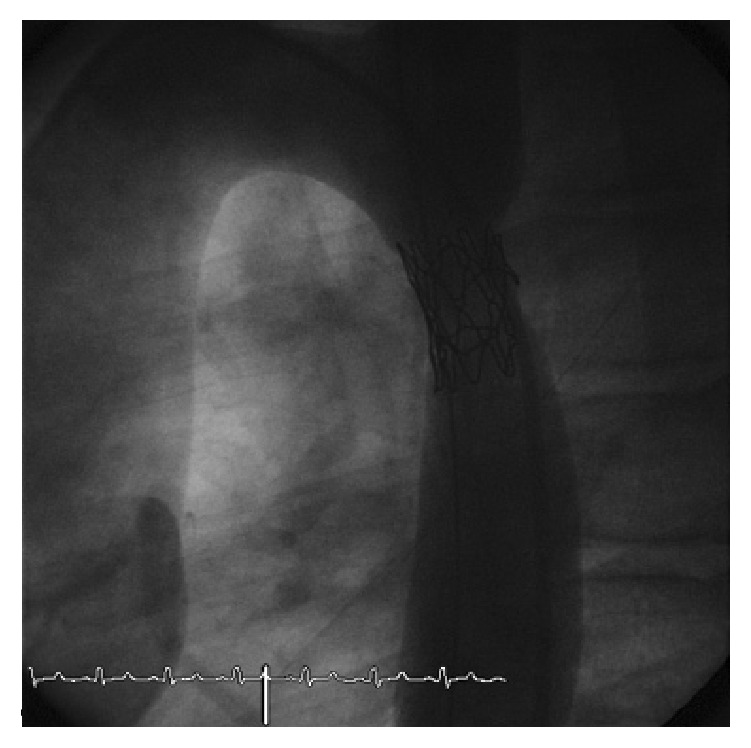
Lateral view: shortening and fracture of the stent after redilatation.

Recent guidelines on endovascular treatment of aortic coarctation have been published by the American Heart Association. However, since at present insufficient data are available to determine the best treatment option in pediatric patients, in this population stenting for aortic coarctation is still a controversial issue [6]. Stent implantation has been developed to neutralize many of the shortcomings of percutaneous balloon angioplasty such as recoil, residual, or recurrent restenosis, possible dissections, and aneurysm due to intimal tear [2]. However, also stenting has some disadvantages, particularly in small growing children: larger sheaths are needed to deliver the stent, which can increase the risk of femoral artery injury. This problem can be minimized using staged dilation: initially low profile balloon is deployed and then the stent is expanded with a larger balloon till the target size is reached. This technique is more demanding and longer [7]. Furthermore the larger use of covered stents, which need 2F larger sheaths, could increase the risk of femoral artery complications, especially in smaller patients. Another disadvantage is growth-induced narrowing of the stent (as shown in Figure 4). Subsequent redilatations could cause shortening or fracture of the stent (as seen in Figure 5), exposing patients to the risk of dissection and aneurysms. When possible, a stent able to reach the final diameter of an adult aorta (18 to 22 mm) should be implanted and subsequently overdilated to minimize foreshortening and fracture rate, even if this choice could affect the profile and the crimpability of the stent, requiring bigger sheaths [2, 8]. Improvement in stent design and long-term follow-up will help determine the role of stent therapy for aortic coarctation [9].

Other possible solutions to the problem of growth-induced stent narrowing have been proposed, such as the Growth Stent, consisting of two stent halves connected by readsorbable sutures and overstented later with larger stents [8]. Finally, another disadvantage of stenting, which can also affect older patients with unknown consequences, may be the introduction of a noncompliant and nonpulsatile segment in the aorta, which can affect the systolic blood pressure at rest or during exercise in the future [10, 11]. Notwithstanding the limits of this retrospective study, we can state that the use of stents in small children to treat AoCo is effective immediately and shortly after the procedure. The main adverse events of stent implantation in smaller children were stenosis recurrence due to growth of the stented vessel and femoral artery injury.

Further studies on larger case series and technological advances in terms of improved materials and techniques could be helpful in the selection of candidates for this procedure.

## 5. Conclusions

In our experience, stenting for aortic coarctation can be considered a feasible but challenging therapeutic option in pediatric patients, in agreement with the results obtained in other studies [12, 13]. We did not find significant differences in the rate of success immediately after the procedures between pediatric and adolescent patients. Also considering the limitations of our study, femoral access-related immediate injury or early thrombosis seemed to occur more frequently in younger patients. Our follow-up data showed that younger patients, due to vessel growth, needed more commonly redilatation, which is often burdened by stent fracture and shortening. Accurate case selection is necessary to evaluate if endovascular treatment is preferable to surgery for each of these patients. Collection of further clinical and imaging data will be helpful to identify the best treatment option for children aged between 12–18 months and 11 years. Long-term follow-up with clinical evaluation, color-Doppler echocardiography, MRI, and stress test is mandatory, because adverse events can occur even many years after the procedure, impacting on the future therapeutic perspectives of these patients.

## Figures and Tables

**Table 1:** Main characteristics of enrolled patients.

	All	Group A	Group B	*p* value
	*N* = 34	*N* = 17	*N* = 17	
Males, *n* (%)	24 (70.6)	11 (64.7)	13 (76.5)	0.71
Age at procedure (yrs)				
Mean ± SD	11.3 ± 3.7	8.2 ± 2.3	14.3 ± 1.7	≤0.0001
Median (range)	11.5 (3; 18)	9 (3; 11)	14 (12; 18)	
Weight (Kg)				
Mean ± SD	40.6 ± 19.4	25.3 ± 4.9	55.9 ± 15.9	≤0.0001
Median (range)	31 (17; 92)	26 (17; 30)	52 (32; 92)	
Native AoCo, yes *n* (%)	23 (67.6)	10 (58.8)	13 (76.5)	0.46
Recurrent AoCo, yes *n* (%)	11 (32.4)	7 (41.2)	4 (23.5)	0.46
Systolic BP (mmHg), mean ± SD	132 ± 18	127 ± 12.5	137 ± 21	0.11
Diastolic BP (mmHg), mean ± SD	71 ± 15	69 ± 12.5	72 ± 17.4	0.65
DP pre (mmHg), mean ± SD	41.41 ± 14.7	43.67 ± 12	39.41 ± 16.8	0.42
DP post (mmHg), mean ± SD	1.61 ± 3	1.67 ± 3.1	1.56 ± 3	0.92
Palmaz Stent, yes *n* (%)	5 (14.7)	4 (23.5)	1 (5.9)	0.33
Bare CP stent, yes *n* (%)	14 (41.2)	5 (29.4)	9 (52.9)	0.29
Covered CP stent, yes *n* (%)	17 (50)	8 (47.1)	9 (52.9)	1

**Table 2:** Late adverse events and follow-up data.

	Group A	Group B	*p* value
	*N* = 17	*N* = 17	
	*N* (%)		
Restenosis	5 (29.4)	1 (5.9)	0.17
Stent fracture	4 (23.5)	1 (5.9)	0.33
Aneurysms after redilatation	1 (5.9)	0	1
Stent migration	1 (5.9)	2 (11.8)	1
Femoral artery stenosis/occlusion	2 (11.8)	1 (5.9)	1
Dissection	0	1 (5.9)	1
Stress test	6 (35.3)	6 (35.3)	0.72
Hypertensive stress response	3 (17.6)	1 (5.9)	0.60

	Mean ± SD		
Systolic blood pressure at 1 year	120 ± 9	126.7 ± 16	0.22
Systolic blood pressure at 5 years	128 ± 16.5	128 ± 11	1
Systolic blood pressure at 10 years	116 ± 7	128 ± 3	0.06
